# Comparison of Antiplatelet Effects of Phenol Derivatives in Humans

**DOI:** 10.3390/biom12010117

**Published:** 2022-01-12

**Authors:** Marcel Hrubša, Raúl Alva, Mst Shamima Parvin, Kateřina Macáková, Jana Karlíčková, Jaka Fadraersada, Lukáš Konečný, Monika Moravcová, Alejandro Carazo, Přemysl Mladěnka

**Affiliations:** 1 Department of Pharmacology and Toxicology, Faculty of Pharmacy in Hradec Králové, Charles University, Ak. Heyrovského 1203, 50005 Hradec Králové, Czech Republic; hrubsam@faf.cuni.cz (M.H.); alvar@faf.cuni.cz (R.A.); fadraerj@faf.cuni.cz (J.F.); konecnylu@faf.cuni.cz (L.K.); moravcovamo@faf.cuni.cz (M.M.); mladenkap@faf.cuni.cz (P.M.); 2 Department of Pharmacognosy, Faculty of Pharmacy, Charles University, Ak. Heyrovského 1203, 50005 Hradec Králové, Czech Republic; parvins@faf.cuni.cz (M.S.P.); macakovak@faf.cuni.cz (K.M.); 3 Department of Pharmaceutical Botany, Faculty of Pharmacy in Hradec Králové, Charles University, Ak. Heyrovského 1203, 50005 Hradec Králové, Czech Republic; karlickova@faf.cuni.cz

**Keywords:** platelet, 4-methylcatechol, flavonoid, aggregation, whole blood, catechol

## Abstract

Flavonoids are associated with positive cardiovascular effects. However, due to their low bioavailability, metabolites are likely responsible for these properties. Recently, one of these metabolites, 4-methylcatechol, was described to be a very potent antiplatelet compound. This study aimed to compare its activity with its 22 close derivatives both of natural or synthetic origin in order to elucidate a potential structure–antiplatelet activity relationship. Blood from human volunteers was induced to aggregate by arachidonic acid (AA), collagen or thrombin, and plasma coagulation was also studied. Potential toxicity was tested on human erythrocytes as well as on a cancer cell line. Our results indicated that 17 out of the 22 compounds were very active at a concentration of 40 μM and, importantly, seven of them had an IC_50_ on AA-triggered aggregation below 3 μM. The effects of the most active compounds were confirmed on collagen-triggered aggregation too. None of the tested compounds was toxic toward erythrocytes at 50 μM and four compounds partly inhibited proliferation of breast cancer cell line at 100 μM but not at 10 μM. Additionally, none of the compounds had a significant effect on blood coagulation or thrombin-triggered aggregation. This study hence reports four phenol derivatives (4-ethylcatechol, 4-fluorocatechol, 2-methoxy-4-ethylphenol and 3-methylcatechol) suitable for future in vivo testing.

## 1. Introduction

Flavonoids are widely present in a diet of plant origin and have been linked to a lower risk of cardiovascular diseases development. The precise mechanism of this finding still needs, however, to be elucidated [1,2,3,4]. In recent years, research has suggested that this effect is not caused by flavonoids themselves, but rather by their metabolites formed by human gut microflora. These bacteria break down parent flavonoids with low bioavailability to smaller compounds, that reach far higher plasma concentrations [5,6].

Following this hypothesis, several research groups, including ours, have found that some of these small phenolic compounds are able to inhibit platelet aggregation in concentrations achievable in human plasma and may therefore modulate the overall hemodynamics and hence possess positive cardiovascular protective effects [7,8,9]. One of these compounds, 4-methylcatechol, was shown to be a very strong antiplatelet compound with an approximately ten times higher potency than clinically used antiplatelet drug, acetylsalicylic acid (ASA) [7]. There is an ongoing search for new antiplatelet drugs with novel mechanism(s) of action since actual pharmacotherapeutic tools face important issues. The major problems are (1) ASA has a relatively high degree of resistance, and (2) other clinically used drugs in outpatient practice (adenosine diphosphate (ADP)-receptor antagonists) are also not always usable due to potential interactions and serious side effects [10,11,12,13,14,15]. 4-methylcatechol, a promising candidate for future preclinical testing, apparently exerts its effect through a different mechanism, likely based on interaction with calcium [7]. We selected a total of 22 compounds structurally closely related to 4-methylcatechol to assess a potential structure–activity relationship regarding the antiplatelet activity. To the best of our knowledge, a study with a similar aim has not been performed yet. Cresols are the only compounds that were examined in terms of antiplatelet effects in addition to pyrocatechol and 4-methylcatechol. Both *m*- and *o*-cresol inhibited platelet aggregation [16]. Their mechanism of action was through inhibition of cyclooxygenase (COX) activity, logically suppressing thromboxane A_2_ (TXA_2_) production and, consequently, platelet aggregation. It should however be mentioned that these compounds are proven pollutants and toxic compounds and hence not feasible candidates for therapy [17,18]. For this reason, this study aimed not only to select the most potent antiplatelet derivative of 4-methylcatechol, but also to investigate the potential toxicity of the tested compounds.

## 2. Materials and Methods

### 2.1. Blood Donors

Blood from 20 non-smoking, young (aged from 22 to 45 years, median 27), healthy volunteers was collected by venipuncture into plastic tubes containing heparin sodium (170 IU/10 mL; Zentiva, Prague, Czech Republic). No medication had been taken by volunteers 14 days prior blood collection and no alcohol had been consumed 12 h before the blood draw. The protocol was approved by the Ethics Committee of the Faculty of Pharmacy, Charles University on May 31, 2019.

### 2.2. Chemicals

4-ethylguaiacol (2-methoxy-4-ethylphenol, W243604), o-toluidine (185426), 4-methylcatechol (M34200), 3-methoxycatechol (M13203), 1,2-dimethoxybenzene (140155), 2-methoxy-4-methylphenol (302880), 2,4-dimethoxytoluene (292559), 4-allyl-1,2-dimethoxybenzene (284424), o-cresol (685700), pyrocatechol (C9510), 3,5-dichlorocatechol (545899), 4,5-dichlorocatechol (547093), 4-*tert*-butylcatechol (19671) 4-nitrocatechol (N15553) and 2-aminophenol (95-55-6) were purchased from Sigma-Aldrich (Sigma-Aldrich, Sant-Louis, MO, USA). 3-aminocatechol (A603283), 4-aminocatechol (A603280), 4-etylcatechol (E901760), 3-fluorocatechol (F588825), 4-fluorocatechol (F588828), 4-chlorocatechol (C381100), 3-isopropylcatechol (I821995) and 3-methylcatechol (M290505) were purchased from Toronto Research Chemicals (Toronto, ON, Canada). All compounds were solved in dimethylsulfoxide (DMSO) unless otherway specified.

DMSO, fetal bovine serum, antibiotics (penicilin/streptomycin) and bovine insulin were purchased from Sigma-Aldrich (Sant-Louis, MO, USA).

### 2.3. Aggregometry

A total of 300 µL of whole blood was diluted with the same volume of 0.9 % sodium chloride (B.Braun, Melsungen, Germany) preheated to 37 °C and incubated with 5 µL of a tested compound dissolved in DMSO (Penta, Prague, Czech Republic) at a final concentration of 0.8% for 3 min at 37 °C. Platelet aggregation was then induced by addition of collagen (Diagnostica a.s., Czech Republic), arachidonic acid (AA) or Thrombin Receptor Activating Peptide (TRAP) (Roche Holding AG, Basel, Switzerland) and monitored for 6 min. The dose of inducer was initially set to the minimal concentration which caused maximal aggregation. The second calibration was carried out by use of standards, 4-methylcatechol in the case of AA- and collagen-triggered aggregation (Sigma-Aldrich) or vorapaxar for thrombin aggregation (Selleck Chemicals GmbH, Munich, Germany). The final concentrations of inducers were in the range of 0.16–2.42 µg/mL for collagen, 25–196 µM for AA and 0.8–9.8 µM for TRAP. For comparison, also the clinically used standard ASA was employed.

### 2.4. Cell Culture

MCF-7/S0.5 human breast cancer cell line adapted to low-sera conditions (ECACC, Porton Down, Salisbury, UK) was used. Cells were maintained at 37 °C and 5% CO_2_ and were passaged once a week. Dulbecco’s Modified Eagle Medium/Nutrient Mixture F-12 (DMEM/F-12; Thermo Fisher, Waltham, MA, USA) supplemented with FBS Charcoal Stripped, antibiotics (penicillin/streptomycin) and insulin, according to cell provider instructions, was used as the growth medium. Cells were used in passages between 7 and 12.

### 2.5. Cytotoxicity Assays

#### 2.5.1. CellTiter 96^®^ AQueous One Solution Cell Proliferation Assay

Cell viability assay was performed using CellTiter 96^®^ AQueous One Solution Cell Proliferation Assay (Promega, Madison, WI, USA). MCF-7 cells were seeded at a density of 80 × 10^3^ cells per well in a 96-well plate. Cells were exposed to the compounds and incubated at 37 °C with CO_2_ 5% for 48 h prior tetrazolium reagent [3-(4,5-dimethylthiazol-2-yl)-5-(3-carboxymethoxyphenyl)-2-(4-sulfophenyl)-2H-tetrazolium] addition (20 µL/well). DMSO at a final concentration of 0.1% (as it is not associated with toxicity) was used as a negative control (set to 100% viability), and sodium dodecyl sulfate (SDS 10%) as positive control. After incubation period CellTiter reagent was added to the cells and further incubated for 3 h. After this time, absorbance was recorded at a wavelength of 490 nm by use of a microplate reader (Hidex Oy, Turku, Finland). All samples were performed in triplicates and repeated at least three times.

#### 2.5.2. Release of Lactate Dehydrogenase (LDH)

These experiments were analogous to our previous experiments with lysis of erythrocytes [19]. Human erythrocyte suspension was obtained by firstly centrifuging whole blood at 2700× *g* for 10 min and discarding the supernatant. Afterward, double volume of saline preheated to 37 °C was added to remaining erythrocytes and sample was again centrifuged at 2700× *g* for 10 min and supernatant was removed. This step was performed twice. The obtained erythrocytes were then diluted 10 times with a solution of 1 mM glucose in saline. Heparin at a final concentration of 10 IU/mL was added to prevent clotting. In this way prepared human erythrocyte suspension was incubated with tested compounds dissolved in DMSO (the final concentration of DMSO was 1%) or saline at a concentration of 50 μM for 4 h at 37 °C. A solution of copper in saline at a final concentration of 500 μM was used as a positive sample (lysis of erythrocytes). The sample was then centrifuged at 1950× *g* for 10 min and 250 μL of the supernatant obtained was used to determine the LDH activity, a marker of cellular lysis. The remaining supernatant was discarded and replaced with a lysis buffer (2 mM EDTA, 1 mM 1,4-dithiothreitol, 1% TRITON-X 100, 0.1 M phosphate buffer of pH 7.8) in the same amount as total supernatant volume. Each sample was thoroughly vortexed and left for 20 min at room temperature to achieve lysis of the remaining erythrocytes. Afterward, samples were centrifuged again for 10 min and 250 μL of supernatant was used to determine LDH activity.

LDH activity was quantified by increase in absorbance caused by the conversion of β-NAD^+^ to β-NADH using a protocol adapted from Chan et al., 2013 [20]. Results were calculated as a percentage of erythrocytes lysed.

### 2.6. Anticoagulation Assay

All compounds were tested for their potential anticoagulation properties using prothrombin time (PT) and activated partial thromboplastin time (aPTT) assays in a semi-automated 4-channel Ceveron^®^ coagulometer (Technoclone, Vienna, Austria). In all experiments, DMSO 1% was used as a negative control since it was used as vehicle for compound solutions. Heparin (0.005 IU/mL and 0.0005 IU/mL, for PT and aPTT respectively) was used as a positive control. A total of 2.1 µL of the compounds were added to 210 µL of commercial normal control plasma at the desired final concentrations. For PT, plasma (100 µL) was incubated at 37 °C for one minute prior to TECHNOPLASTIN-HIS addition (200 µL). For aPTT, incubation of plasma (again 100 µL) with the same amount of DAPTTIN reagent was performed for 2 min prior to addition of 100 µL of 25 mM CaCl_2_. Experiments were performed in duplicates in at least three independent experiments. All reagents and materials for these experiments were purchased from Technoclone.

### 2.7. Statistical Analysis

Results are always presented as means ±SD of at least three independent experiments. Aggregation results were assessed by comparing 95% confidence intervals of fitted curves. One-way ANOVA followed by Dunnett’s multiple comparison test was used to determine the significance of the obtained results in the remaining experiments. All statistical studies were performed using GraphPad v. 9.2.0 (332) software (GraphPad Software, San Diego, CA, USA).

## 3. Results

**Figure 1:**
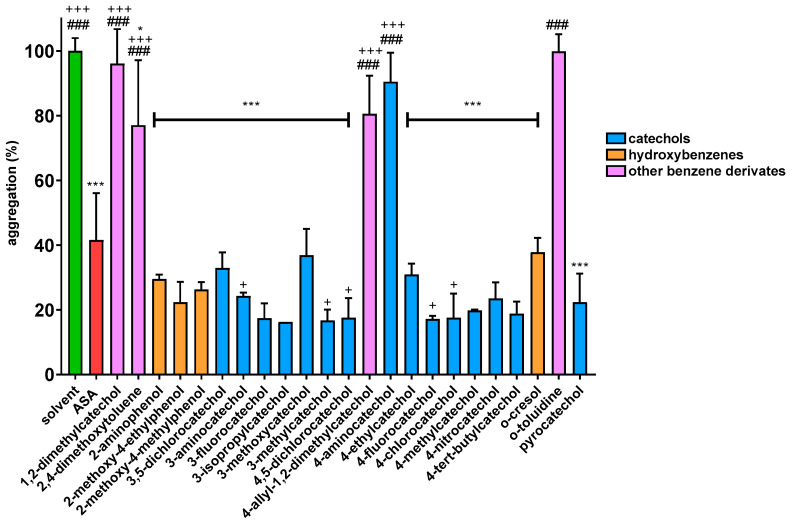
Effect of Phenolic Compounds on AA-induced Aggregation: Results are presented as means with ±SD. * *p* ˂ 0.05 vs. DMSO; *** *p* ˂ 0.001 vs. DMSO; ^+^ *p* ˂ 0.05 vs. ASA; ^+++^ *p* ˂ 0.001 vs. ASA; ^###^ *p* ˂ 0.001 vs. 4-methylcatechol.

**Figure 2:**
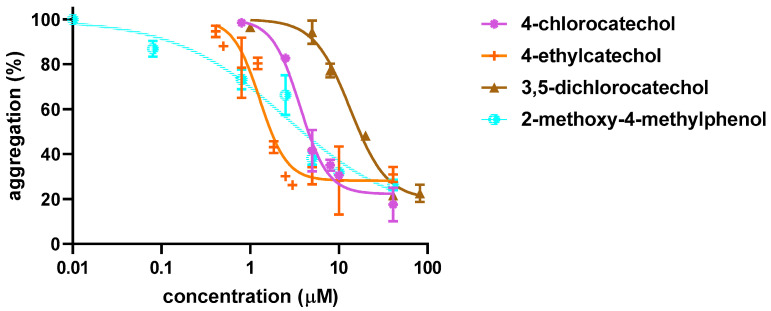
Examples of Dose–response Curves in Arachidonic Acid-triggered Platelet Aggregation. Four compounds with different potencies were intentionally selected. Results show the summary of three independent experiments. Curves were fitted using a non-linear regression method.

**Figure 3:**
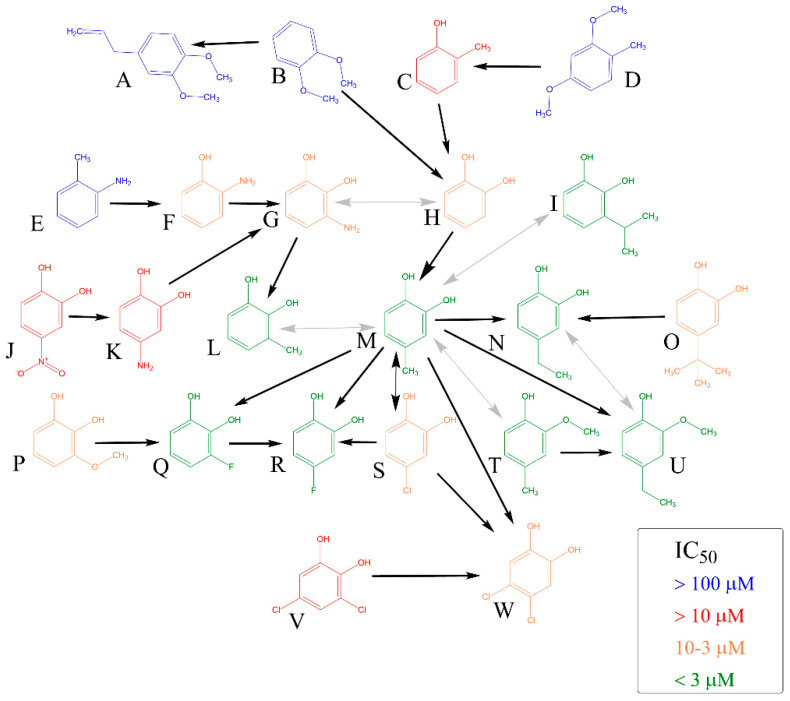
Comparison of Antiplatelet Activity of All Compounds on Arachidonic Acid-platelet Aggregation. The direction of black arrow shows a more potent compound. Analysis was performed by comparing 95% confidence intervals of aggregation curves. Two-way grey arrows mean no statistical difference in the activity. (**A**): 4-allyl-1,2-dimethylcatechol, (**B**): 1,2-dimethylcatechol, (**C**): *o*-cresol, (**D**): 2,4-dimethoxytoluene, (**E**): *o*-toluidine, (**F**): 2-aminophenol, (**G**): 3-aminocatechol, (**H**): pyrocatechol, (**I**): 3-isopropylcatechol, (**J**): 4-nitrocatechol, (**K**): 4-aminocatechol, (**L**): 3-methylcatechol, (**M**): 4-methylcatechol, (**N**): 4-ethylcatechol, (**O**): 4-*tert*-butylcatechol, (**P**): 3-methoxycatechol, (**Q**): 3-fluorocatechol, (**R**): 4-fluorocatechol, (**S**): 4-chlorocatechol, (**T**): 2-methoxy-4-methylphenol, (**U**): 2-methoxy-4-ethylphenol, (**V**): 3,5-dichlorocatechol, (**W**): 4,5-dichlorocatechol.

Firstly, initial screening of all compounds at a concentration of 40 µM on platelet aggregation triggered by AA was performed using human whole blood (Figure 1). This initial testing identified 17 from 22 compounds that were numerically more efficient than our standard ASA and had statistically similar activity to 4-methylcatechol or were even more potent. In the next step, dose–response curves of these efficient compounds were constructed (examples are shown in Figure 2). Their IC_50_ are shown in Table 1. Comparison of the effect of different compounds enabled the creation of a structure–activity relationship scheme (Figure 3).

**Figure 4:**
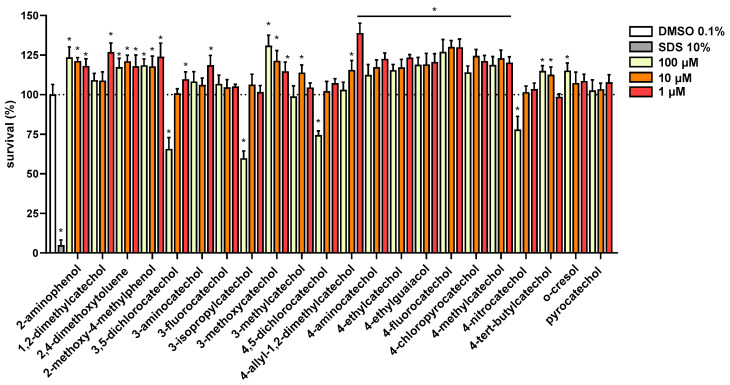
Cytotoxicity of All the Compounds was Tested in MCF7/S0.5 Breast Cancer Cell Line. All compounds were tested in three different concentrations and results are compared to DMSO 0.1%, set to 100% viability. SDS 10% was used as a positive control. All compounds were tested in triplicates at least three times. Statistical significance was established using a one-way ANOVA assay with confidence interval of 95% (* *p* < 0.001). *o*-Toluidin was not tested.

Further, the cytotoxicity of these compounds was tested using LDH assay in human erythrocytes (Figure S1) and CellTiter 96^®^ assay in a human cancer cell line (Figure 4). In both assays, tested compounds showed to be non-toxic with the exception of four compounds (3-isopropylcatechol, 3,5-dichlorocatechol, 4,5-dichlorocatechol and 4-nitrocatechol). The latter four compounds decreased cell proliferation at the highest tested concentration of 100 μM but were not toxic at 10 μM.

As the combination of antiplatelet and anticoagulant activity can be advantageous, also the influence of the compounds on blood coagulation was also tested. However, none of the tested compounds significantly affected coagulation, even at 100 μM, the highest concentration assayed (Figure S2).

**Figure 5:**
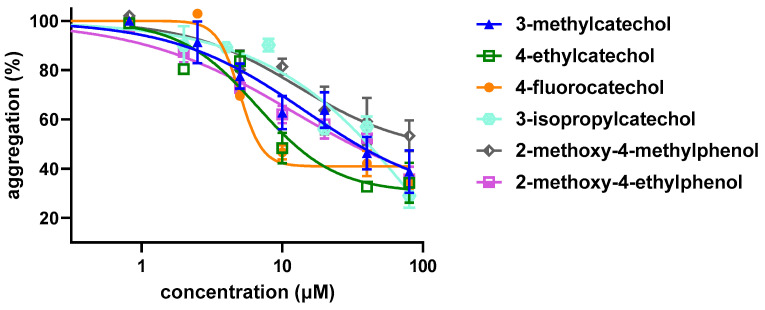
Dose–response Curves of the Most Efficient and Safe Compounds on Collagen-induced Platelet Aggregation. 4,5-dichlorcatechol is not shown since it blocked cell proliferation at 100 μM.

Lastly, the antiplatelet effect of the most active seven derivatives was confirmed on platelet aggregation induced by collagen, the most known physiological inducer (Figure 5, Table 2). Importantly, all compounds demonstrated to have high activity, with a similar effectivity range as 4-methylcatechol (Figure 5). All seven compounds had a numerically better effect than ASA (Table 2). Comparison of 95% confidence intervals of nonlinear fitted aggregatory curves showed three compounds to be significantly more efficient than ASA, namely 4-ethylcatechol, 4-fluorocatechol and 2-methoxy-4-methylphenol.

To see if these compounds can also affect other platelet aggregation pathways, we used thrombin receptor activating peptide (TRAP) to trigger blood aggregation. In line with the results of 4-methylcatechol [7], the tested compounds were not able to inhibit platelet aggregation induced through this pathway in contrast to clinically used antagonist vorapaxar (Figure S3).

## 4. Discussion

Reported data on tested derivatives of 4-methylcatechol from the literature should be very carefully examined since many of them are related to high doses or concentrations. This information includes both positive and negative effects. Positive effects encompass, beyond the known direct antioxidant effect of phenolic compounds [21,22], antiviral and antibacterial activities, enhanced synthesis of endogenous antioxidants, anti-inflammatory and antiplatelet effects. Antiviral activity against encephalomyocarditis virus was however tested only in very high concentrations–IC_50_ of catechol was 0.67 mg/mL (6 mM). 3- and 4-methylcatechols, as well as 3-methoxycatechol and 4-ethylcatechol, were more potent, but the lowest tested concentration was 0.4 mg/mL (approximately 3 mM) [23]. Antibacterial activity was observed for catechol, 4-chlorocatechol as well as 3,5-dichlorocatechol. However, only the latter seems to have usable activity for systemic administration, since it blocked the formation of bacterial colonies at 10 μM [24]. The activation of a key antioxidant nuclear factor Nrf2 was clearly confirmed in many cell lines by a number of catechols (pyrocatechol, 4-methyl, 4-vinyl and 4-ethylcatechol). Methylated catechols were inactive as well as a monohydroxylated phenol (4-ethylphenol). Importantly such effect was observed even in a concentration of 5 μM [25]. Whether an increase in oxidative stress plays a role was not investigated, but hypoxia markedly reduced the effect, and another study found an increase in intracellular oxidative stress after incubation with 3-methyl, 4-methyl and 4-ethylcatechol [23,25]. Pro-oxidation activates this pathway, as was also demonstrated for other structurally different compounds [26] and could be hence responsible for this effect. 2-methoxy-4-ethylphenol (ethylguaiacol) activated the Nrf2 pathway as well, but only concentrations higher than 650 μM were tested [27]. The same compound, however, activated the Nrf2 pathway in another study after its suppression by lipopolysaccharide even in a concentration of 10 μM. Its anti-inflammatory activity was observed at the same concentration [28,29].

Data on antiplatelet effect were reported in addition to pyrocatechol and 4-methylcatechol only for cresols [16] and only indirectly suggested for a row of small phenolic derivatives, in particular with 2,4-dimethoxytoluene core, from *Chamomilla recutita* (L.) Rauschert [30]. In this study, the current knowledge in relation to antiplatelet activity of different synthetic and natural small phenolics was largely extended. Aggregation experiments utilizing AA have revealed several potent antiplatelet compounds. It should be emphasized that their potency was very high, as most of them had IC_50_ values in the range of units of μM concentration. Some of the tested compounds are known metabolites of food polyphenolic compounds. For example, the plasma levels of the pyrocatechol metabolite pyrocatechol-sulfate reached even 12 μM in humans on average, these data might not be only relevant for the development of a novel antiplatelet drug but might also have epidemiological importance [5].

In this study, we also showed, that the presence of at least one hydroxyl group is essential for antiplatelet activity. A good example are dimethoxyderivatives, i.e., 1,2-dimethylcatechol (1,2-dimethoxybenzene) and 4-allyl-1,2-dimethoxybenzene (methyleugenol) since they were inactive towards AA-induced aggregation. In general, none of the benzene derivatives without a free hydroxyl have shown activity in biologically relevant concentration towards AA-induced aggregation (Figure 1).

On the contrary, catecholic compounds substituted with an alkyl chain and one or more halogens possessed significant activity. This confirms that the antiplatelet activity is dependent on ortho-dihydroxy moiety, one of the hydroxyls can, however, be methylated without significant loss of activity. This can be seen in a very similar potency of 4-methylcatechol vs. 2-methoxy-4-methylphenol and 4-ethylcatechol vs. 2-methoxy-4-ethylphenol (Table 1, Figure 3).

Further substitution of the catechol structure, with retaining of ortho-dihydroxy moiety, provided us with more insights on the structure–activity relationship. Branched alkyl substituents clearly diminished the activity, as can be seen in 4-*tert*-butylcatechol. Contrarily, the position of the methyl, or its replacement by ethyl or even isopropyl or fluorine had only little effect on the final effect. The effect of nitrogen was always negative, as both amino and nitro substitutions decreased the potency. It is unclear, if this is based on the size or the character of the substituent, since (a) introduction of a larger chlorine atom clearly decreased the activity in comparison to smaller halogen fluorine, but (b) insertion of two chlorine atoms in positions four and five but not three and five even improved the activity.

Substitution in position three seems to be detrimental in general, but the nature of the substituent played a key role here as well. 3-methylcatechol was one of the most potent antiplatelet compounds, the same is true for 3-fluorocatechol. It should be however emphasized that 3-isopropylcongener together with both dichloro substituted compounds and 4-nitroderivative represent only the partly toxic compounds as they blocked cell proliferation at 100 μM (Figure 4). Anyway, these compounds were safe at 50 μM when incubated with human red blood cells and without an effect when incubated with a selected breast cancer cell line at 10 μM (Figure 4 and Figure S1). Hence this toxicity had no effect in relation to this study. It could be concluded that approximately with increasing size of substituent, a gradual decrease in activity: 3-methylcatechol ˃ 3-fluorocatechol ˃ 3-isopropylcatechol ˃ 3-aminocatechol ˃ 3-methoxycatechol was observed.

Beyond the strong effect, the second crucial feature is the potential safety of these compounds. This is particularly important for this group as some distant derivatives were shown to be genotoxic and/or causing the development of tumors. This is however related in particular to *o*-toluidine and 4-allyl-1,2-dimethoxybenzene (methyleugenol). The former is a very probable human and animal carcinogen able to cause bladder carcinoma when exposed to high doses on a long-term basis [31]. 4-allyl-1,2-dimethoxybenzene elicited liver but also other tumors in rats and mice. Importantly, the measured rat plasma levels in chronic high dose studies were in the range of 4–46 μM (0.66–8.25 μg/mL). Humans are normally exposed to only low doses, and therefore this risk is likely irrelevant under common exposition [32,33]. Regardless, both compounds were inactive or only weakly active in our antiplatelet assay, but they were included for the reason of the structure–activity analysis. There are also some claims that pyrocatechol or its derivatives could also bring some risks [34]. This is however not very likely, since as was mentioned above, catechol is a common dietary metabolite and humans are hence commonly exposed to this compound in relatively high concentrations.

Nonetheless, due to claims of potential toxicity, testing of cytotoxic potential was included. As emphasized above, except for four compounds at a high concentration of 100 μM, they were not toxic to cells. In some cases, they rather had weak proliferative effects. The safety of most catechols is in line with previous studies. Pyrocatechol, 4-methylcatechol, 4-vinylcatechol and 4-ethylcatechol in a concentration of 30 μM were not toxic to three different cell lines and again rather mild proliferative effects were observed in some cases [25]. 2-Methoxy-4-ethylphenol was mildly toxic toward hepatic HepG2 but from a concentration of about 660 μM (100 mg/L). In another study, its toxicity toward human monocytic cell line THP-1 was not observed in concentrations lower than 5 mM [27,28,29]. Additionally, no DNA damage was observed when pyrocatechol, 4-chlorocatechol or 3,5-dichlorocatechol were incubated with plasmids without redox-active metals at 50 μM [35].

This study was planned as an initial selection of the most active and safe compounds for future testing. Hence, it was not able to answer all questions. In particular, it will be needed to detect the mechanism of action, which is neither fully elucidated for 4-methylcatechol [7]. As 4-methylcatechol potently blocked the AA- and collagen-based pathway and had no effect on thrombin, it is well probable that the mechanism of action between the most potent catechols will be the same. Indeed, *o*-cresol also had no effect on the thrombin pathway [16].

## 5. Conclusions

This study has shown that several derivatives of 4-methylcatechol are strong antiplatelet drugs with higher potency than clinically used acetylsalicylic acid. In particular four compounds (4-ethylcatechol, 4-fluorocatechol, 2-methoxy-4-ethylphenol and 3-methylcatechol) seem to be safe, very efficient but future testing including confirmation in in vivo conditions are needed for future development. 

## Figures and Tables

**Table 1:** IC_50_ on Arachidonic Acid-induced Platelet Aggregation.

Compound	IC_50_ (Mean ± SD, μM)
1,2-dimethylcatechol	≈78
2,4-dimethoxytoluene	≈32
2-aminophenol	6.65 ± 1.06
2-methoxy-4-ethylphenol	1.37 ± 0.28
2-methoxy-4-methylphenol	2.57 ± 2.24
3,5-dichlorocatechol	13.49 ± 1.57
3-aminocatechol	3.82 ± 0.50
3-fluorocatechol	1.74 ± 0.17
3-isopropylcatechol	2.28 ± 0.30
3-methoxycatechol	7.92 ± 0.74
3-methylcatechol	1.61 ± 0.17
4,5-dichlorocatechol	3.35 ± 0.92
4-allyl-1,2-dimethylcatechol	≈61
4-aminocatechol	≈21
4-chlorocatechol	3.66 ± 0.29
4-ethylcatechol	1.29 ± 0.14
4-fluorocatechol	1.82 ± 0.21
4-methylcatechol	2.59 ± 0.17
4-nitrocatechol	13.27 ± 1.57
4-*tert*-butylcatechol	7.24 ± 3.75
o-cresol	11.97 ± 5.55
pyrocatechol	4.00 ± 0.45

**Table 2:** IC_25_ on Collagen-Induced Platelet Aggregation.

Compound	IC_25_ (Mean ± SD, μM)
2-methoxy-4-ethylphenol	5.07 ± 4.05
2-methoxy-4-methylphenol	11.54 ± 9.05
3-isopropylcatechol	12.86 ± 21.28
3-methylcatechol	7.15 ± 6.41
4,5-dichlorocatechol	9.86 ± 15.05
4-ethylcatechol	4.61 ± 1.16
4-fluorocatechol	4.72 ± 0.28
4-methylcatechol	2.66 ± 2.21
ASA	26.85 ± 2.25

## Supplementary Materials

The following are available online at https://www.mdpi.com/article/10.3390/biom12010117/s1, Figure S1: Effect of the tested compounds on human erythrocytes. Figure S2: Effect of tested compounds on blood coagulation. Figure S3: Effect of selected most active compounds on TRAP-induced platelet aggregation.
