# Supplementary material for: Comparison of Antiplatelet Effects of Phenol Derivatives in Humans
Source: Biomolecules. 2022 Jan 12;12(1):117. doi: 10.3390/biom12010117 (PMC8774223; doi:10.3390/biom12010117)
Supplement: Supplementary file 1 [file biomolecules-12-00117-s001.zip › biomolecules-1529956-supplementary.pdf]

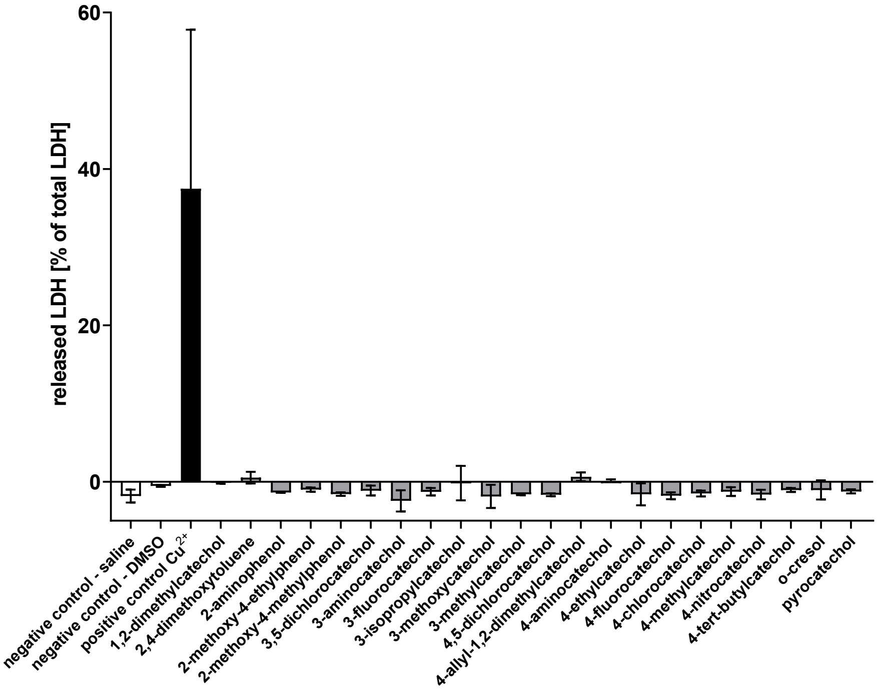

Figure S1. Effect of the tested compounds on human erytrocytes. Tested compounds or their solvents DMSO or saline were incubated with human purified erytrocytes for 4 h at 37 °C. The lysis of erytrocytes was measured by release of lactate dehydrogenase. Cupric ions at a final concentration of 500 μM was used as a positive control. Most compounds were dissolved in saline with the exception of 3-isopropylcatechol, 4-allyl-1,2-dimethylcatechol, 1,2-dimethylcatechol and 2,4-dimethoxytoluene that were dissolved in DMSO. *o*-Toluidin was not tested in this assay.

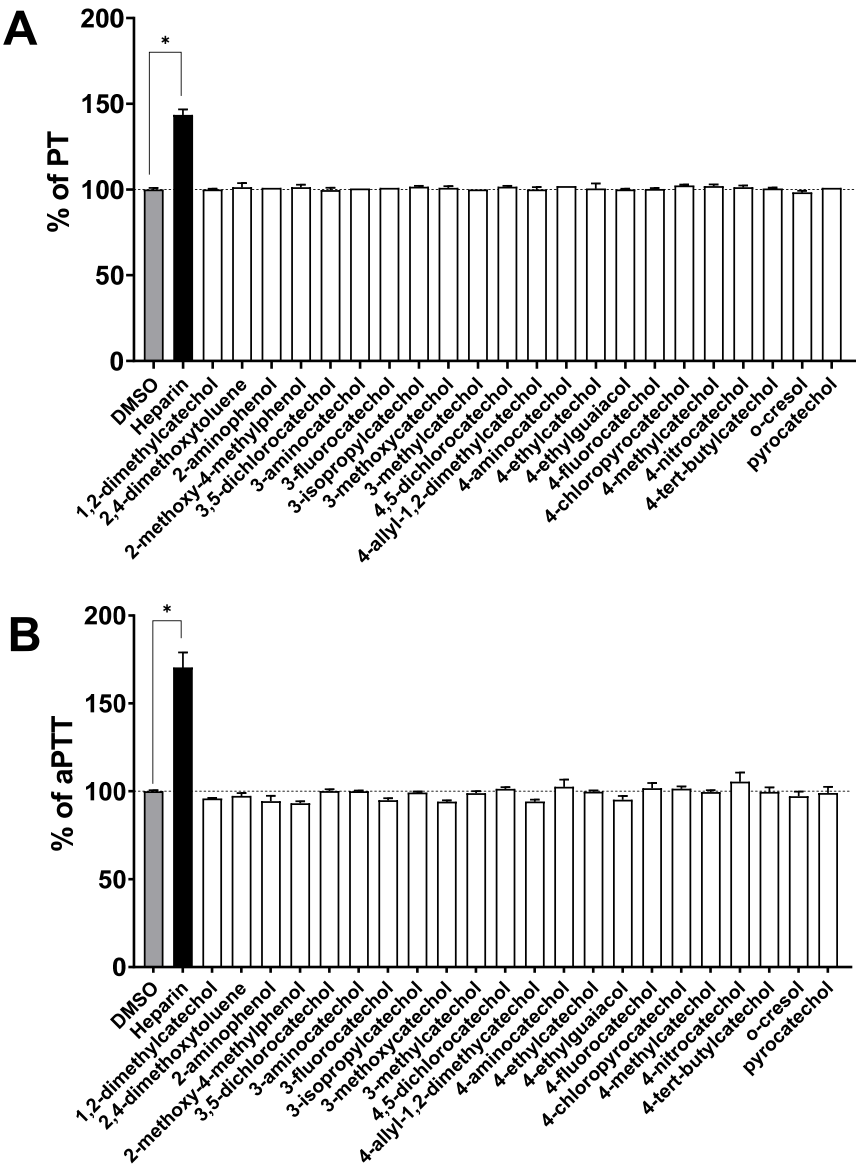

Figure S2. Effect of tested compounds on blood coagulation. Prothrombin time (PT) and activated partial thromboplastin time (aPTT) values of the tested compounds at final concentration of 100 μM were determined using Ceveron® coagulometer. For both methods, DMSO 1% was used as vehicle (an also as a negative control) and heparin as positive control at a final concentration 0.005 IU/mL and 0.0005 IU/mL for PT and aPTT, respectively. *$p < 0.001$.

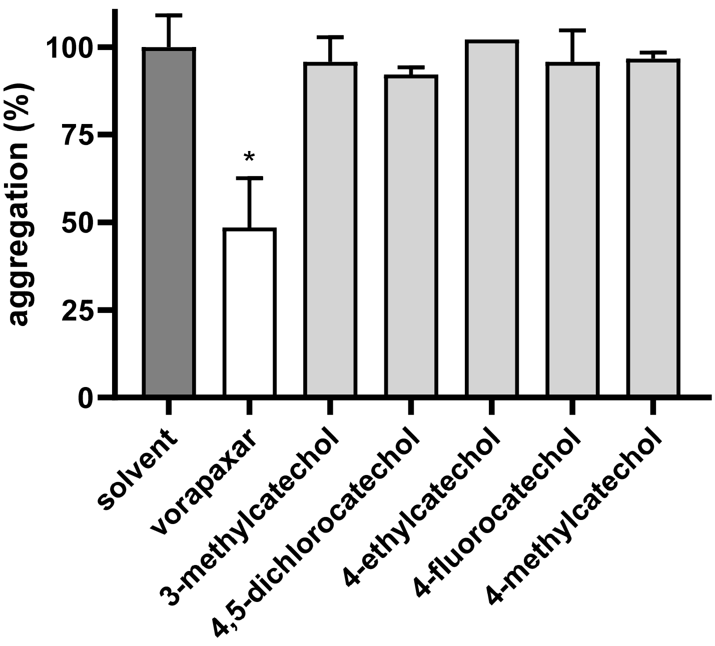

Figure S3. Effect of selected most active compounds on TRAP induced platelet aggregation. Blood aggregation was triggered by adding TRAP, concentration of tested compounds was 250 μM, except for vorapaxar which was investigated at 1.5 μM. Values represented as means with SD. *p < 0.05 vs. DMSO.
